# Dietary inflammatory index and the risks of non-alcoholic fatty liver disease: a systematic review and meta-analysis

**DOI:** 10.3389/fnut.2024.1388557

**Published:** 2024-07-25

**Authors:** Xingfen Zhang, Jiale Ruan, Yujing He, Anyi Xu, Yingying Fang, Qiufeng Zhang, Lihu Gu, Xingchen Liu

**Affiliations:** ^1^Department of Liver Disease, Ningbo No. 2 Hospital, Ningbo, Zhejiang, China; ^2^The First Clinical Medical College, Zhejiang Chinese Medical University, Hangzhou, Zhejiang, China; ^3^Sir Run Run Shaw Hospital, Zhejiang University School of Medicine, Hangzhou, Zhejiang, China; ^4^Department of General Surgery, Ningbo No. 2 Hospital, Ningbo, Zhejiang, China; ^5^Intensive Care Unit, Ningbo No. 2 Hospital, Ningbo, Zhejiang, China

**Keywords:** dietary inflammatory index, non-alcoholic fatty liver disease, inflammation, diet, meta-analysis

## Abstract

**Background:**

Previous studies have suggested a correlation between dietary inflammatory potential and non-alcoholic fatty liver disease (NAFLD). Therefore, the study aimed to investigate the association between dietary inflammatory potential, measured by the dietary inflammation index (DII), and NAFLD.

**Methods:**

From establishing the database to June 2023, a systematic search of PubMed, Web of Science, Embase and Cochrane Library were performed to identify relevant observational studies. These studies reported a correlation between DII and NAFLD. The meta-analysis used odds ratio (OR) with 95% confidence intervals (CI) to evaluate the relationship between DII and NAFLD.

**Results:**

Eight studies were included in this meta-analysis after excluding irrelevant records. A summary of the results from the included studies showed that the risk of NAFLD was higher in those exposed to higher DII (OR = 1.26, 95%CI 1.12 to 1.40, *p* < 0.001), with a high degree of heterogeneity (I^2^ = 85.7%, *p* < 0.001). When DII was divided into 3 tertiles from low to high for comparison, the results showed that the risk of NAFLD was higher in Tertile 2 (T2) population compared to the Tertile 1 (T1) population (OR = 1.75, 95%CI 1.20 to 2.54, *p* < 0.005). The risk of NAFLD was significantly higher in Tertile 3 (T3) compared to the T1 population (OR = 3.07, 95%CI 1.63 to 5.77, *p* = 0.001).

**Conclusion:**

The results suggest that high DII is associated with an increased risk of NAFLD, and conversely, low DII is associated with a decreased risk of NAFLD.

**Systematic Review Registration:**

The study complies with the Preferred Reporting Items for Systematic Reviews and Meta-Analyses (PRISMA) guidelines and is registered on PROSPERO (CRD42023455013).

## Introduction

Over the past 40 years, non-alcoholic fatty liver disease (NAFLD) has become the most common chronic liver disease. The incidence rate of NAFLD ranges from 13.5% (Africa) to 31.8% (Middle East), with a world average incidence rate of 20%. Obesity, type 2 diabetes mellitus (T2DM) and hyperlipidemia are the major risk factors for NAFLD. Notably, the incidence of NAFLD in obese patients has reached a staggering 80%. In terms of the dangers of NAFLD, although less than 10% of patients will develop liver fibrosis or hepatocellular carcinoma within 10–20 years (1–3), the dangers cannot be ignored given the large number of patients. Moreover, based on the increasing obesity rate in the population and the extremely high incidence rate of NAFLD in the obese population, the economic burden of people on NAFLD increases year by year.

Inflammation, as a response of the body to tissue damage, involves massive recruitment of inflammatory factors and inflammation-associated cells. The pro-inflammatory mediators tumor necrosis factor-α (TNF-α), interleukin-6 (IL-6), interleukin-8 (IL-8), interleukin-10 (IL-10) and interleukin-1β (IL-1β) contribute to the recruitment and activation of Kupffer cells, whilst the massive activation of Kupffer cells eventually leads to NAFLD (3). Besides, several studies have shown that specific dietary components are associated with inflammation. A recently published meta-analysis suggested that the Mediterranean diet (MD), as a dietary pattern with low inflammatory potential, significantly reduces the risk of NAFLD compared to other dietary patterns (4). Generally speaking, the MD is characterized by a diet rich in plant foods (vegetables, grains, legumes, fruits, and nuts), moderate amounts of fish and poultry, small amounts of red meat, and foods with a low degree of processing (5, 6). Additionally, high-sugar and high-fat dietary patterns have been shown to cause the body to produce high levels of inflammatory factors which can increase the risk of NAFLD or even exacerbate a patient’s pre-existing liver disease (7, 8).

The dietary inflammation index (DII), a tool to assess the ability of diet to influence inflammatory processes, was first proposed by researchers at the University of South Carolina in 2009 and has been continuously updated and refined since then (9). The revised version of the DII currently in common use is based on literature on inflammation and diet published up to 2010. The DII categorizes an individual’s inflammatory potential from maximally anti-inflammatory to maximally pro-inflammatory based on the potential of the diet. A higher DII indicates higher pro-inflammatory potential and a lower DII indicates higher anti-inflammatory potential. The DII consists of 45 food parameters, including 36 anti-inflammatory foods (fiber, alcohol, monounsaturated fatty acids, polyunsaturated fatty acids, omega 3, omega 6, niacin, thiamin, riboflavin, vitamin B6, zinc, magnesium, selenium, vitamin A, vitamin C, vitamin D, vitamin E, folic acid, beta carotene, anthocyanidins, flavan3ols, flavonols, flavanones, flavones, isoflavones, garlic, ginger, onions, thyme, oregano, saffron, turmeric, rosemary, eugenol, caffeine, and tea) and 9 pro-inflammatory foods (energy, carbohydrates, proteins, total fat, trans fat, cholesterol, vitamin B12, saturated fatty acids and iron) (9, 10). This meta-analysis is based on the results of published studies that aims at investigating the association between the DII and the risk of NAFLD.

## Methods

The study complies with the Preferred Reporting Items for Systematic Reviews and Meta-Analyses (PRISMA) guidelines (11) and is registered on PROSPERO (CRD42023455013).

### Search strategy and study selection

From the inception of the database to June 2023, studies on the relationship between the DII exposed to NAFLD were searched in the electronic database including PubMed, Web of Science, Embase and Cochrane Library. The following was related terms for search use: (non-alcoholic fatty liver disease OR non alcoholic fatty liver disease OR NAFLD OR nonalcoholic fatty liver disease OR nonalcoholic fatty liver OR nonalcoholic fatty livers OR nonalcoholic steatohepatitis OR nonalcoholic steatohepatitides OR metabolic dysfunction-associated steatotic liver disease OR MASLD OR metabolic dysfunction-associated steatohepatitis OR MASH) AND (dietary inflammatory index OR inflammatory diet OR anti-inflammatory diet OR dietary score). The reference lists of relevant articles were searched by 2 researchers to avoid omissions.

Articles were independently assessed by 2 researchers as to whether they met the inclusion and exclusion criteria. Studies will be considered for inclusion if they meet each of the following inclusion criteria: (i) patients in the exposure group have a higher DII and patients in the non-exposed group experienced a lower DII; (ii) a correlation between DII and NAFLD is reported; (iii) the study provided an outcome of DII and NAFLD; and (iv) the study is an observational study (cohort study, case–control study and cross-sectional study) or a randomized controlled trial (RCT).

At the same time, articles meeting one of the following exclusion criteria would be excluded: (i) the full text cannot be obtained; (ii) articles were not written in English; (iii) the data was not available; and (iv) for the same cohort, the most comprehensive or latest article would be included.

### Data extraction and quality assessment

The purpose of this study was to explore the risks of different DII-causing NAFLD in the population. NAFLD is an overarching term defined as a liver disease in which ≥5% of hepatocytes display macrovesicular steatosis in individuals who drink little or no alcohol (defined as <20 g/day for women and < 30 g/day for men) in the absence of readily identified alternative cause of steatosis (eg., medications, starvation, and monogenic disorders). The spectrum of disease includes nonalcoholic fatty liver (NAFL) and nonalcoholic steatohepatitis (NASH) (1, 12). According to the Delphi consensus statement, from 2023, the American Association for the Study of Liver Diseases (AASLD), the European Association for the Study of the Liver (EASL), and the Latin American Association for the study of the liver (ALEH) have recommended the use of metabolic dysfunction-associated steatotic liver disease (MASLD) as an alternative to NAFLD (13, 14). However, in our research, we followed the previous convention of using the term NAFLD.

DII was defined as an indicator to assess the effects of diet on the inflammatory response of the human body. The DII proposed by Shivappa et al. in 2014 included 45 different dietary factors (food parameters consisting of nutrients and whole foods) and their pro-inflammatory or anti-inflammatory effects (9). For the range of DII obtained from the included studies in different populations, we divided the DII into three different quartiles in ascending order. The populations were divided into three quartiles according to different DII, namely Tertile 1 (T1), Tertile 2 (T2), and Tertile 3 (T3).

The research data was extracted independently by 2 investigators. The following information was extracted using a pre-determined data collection form: (i) authors; (ii) year of publication; (iii) country; (iv) research cohort; (v) study duration; (vi); follow-up period (vii) participant profiles; (viii) measurement tool; (ix) adjusting factors; and (x) relevant data.

The quality of cohort and case–control studies was assessed based on the Newcastle-Ottawa Quality Assessment Scale, which consists of 3 quality parameters: selection (4 points), comparability (2 points) and results (3 points), with a score of 6 and above indicating high quality (15). Meanwhile, the quality of the cross-sectional study was assessed according to the Agency for Healthcare Research and Quality (AHRQ) cross-sectional research evaluation standard (16). There are 11 criteria, and a score of 8 points and above was considered high quality. Finally, the quality of included RCTs was assessed using the Cochrane collaboration recommendations (17).

### Statistical analysis

The study used odds ratio (OR) with 95% confidence intervals (CI) to evaluate the relationship between DII and NAFLD. The chi-square test was used to determine heterogeneity, with I^2^ values ≤30% indicating low heterogeneity; I^2^ values between 30 and 60% indicating medium heterogeneity; the I^2^ values ≥60% indicating high heterogeneity (18). Due to the potential heterogeneity among the inclusion studies, the study used a randomized model to improve the credibility of the results. Meanwhile, publication bias was assessed by using the Begg’s test (19). In addition, the stability of the results was evaluated by sensitivity analyses. The two-tailed *p* test was used to define a *p*-value of less than 0.05 as statistically significant. All analyses were performed using STATA version 12.0.

## Results

### Study selection and data extraction

**Figure 1:**
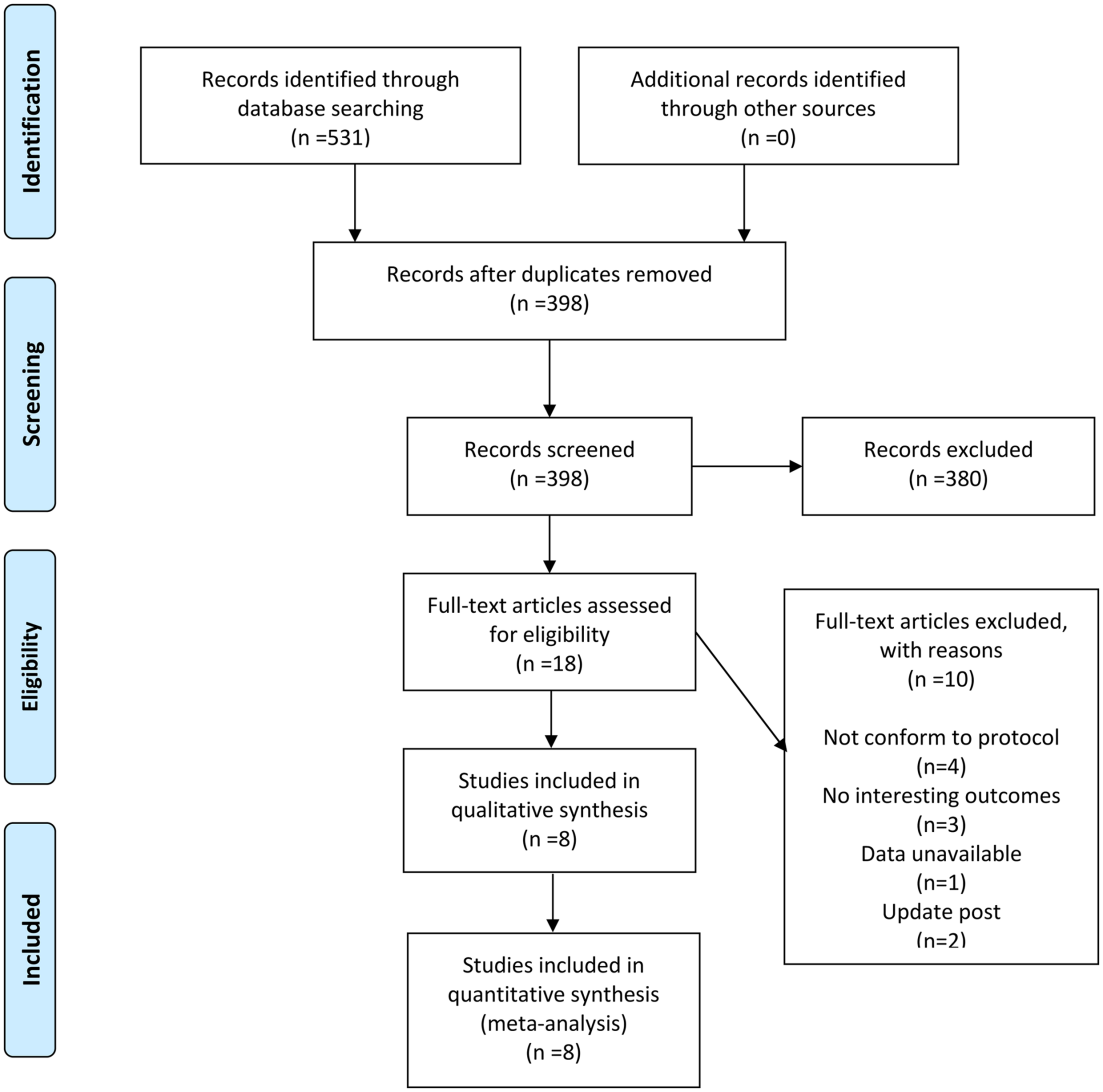
Flow diagram describing inclusion and exclusion criteria.

The entire process of finding and screening for the studies is presented in Figure 1. According to the title, abstract and full manuscript, a hierarchical approach was applied to assess the relevance of the research. First of all, 531 relevant records were identified through the pre-determined search strategy. A total of 398 studies were identified after eliminating duplicates. Then, 380 were excluded after a review of titles and abstracts. Next, of the 18 articles selected, 10 were not included for the following reasons: (i) not comply with the protocol; (ii) no interesting outcomes; (iii) data unavailable; and (iv) update post. Finally, 8 articles that conformed to the inclusion criteria were included in this meta-analysis.

### Study characteristics

A total of 2 million individuals were included in the 8 studies selected for meta-analysis. Table 1 shows the basic information extracted from all 8 included studies (20–27). All studies were observational studies and published between 2018 and 2023. 2 of them were case–control studies (20, 25), the other 2 were cohort studies (21, 24) and 4 were cross-sectional studies (22, 23, 26, 27). The duration of the experiment ranged from 6 months to 144 months. Exposure tools comprised the food frequency questionnaire (FFQ) and Oxford WebQ. The DII consists of 45 dietary parameters. Among the eight included studies, two studies used 31 dietary parameters, two studies used 26 dietary parameters, one study used 18 dietary parameters, and the remaining study was not mentioned. In addition, there were 7 to 8 pro-inflammatory dietary parameters (such as Vitamin B12, iron and protein) and 11 to 23 anti-inflammatory dietary parameters (such as Vitamin A, Vitamin C and Vitamin D). Most studies were adjusted for covariates that may have a significant impact on NAFLD, including, but not limited to: age, sex, body mass index (BMI), education, smoking, alcohol consumption, physical activity, obesity, and ethnicity. The comprehensive set of covariates for which adjustments were made are described in Table 1.

**Table 1:** Characteristics of included observational studies in the meta-analysis.

Author, year	Country	No. of participants	Duration of experiment (year)	Exposure assessment	Adjust parameters	Study design
Moradi, F. 2022	Iran	240	Sept. 2019–Feb. 2020	FFQ	Age, sex, education, physical activity, BMI and SES, energy intake, taking medication, and supplements	Case–control study
Petermann-Rocha, F. 2023	UK	171,544	2006–2010	Oxford WebQ	Age, sex, poverty, ethnicity, obesity, blood glucose, hypertension, HDL, triglyceride, inflammatory diseases, smoking and physical activity	Cohort study
Vahid, F. 2018	Iran	999	Jan. 2015–Dec. 2015	FFQ	Age, BMI, LDL, TG, AST/ALT, education, smoking, alcohol consumption, and blood glucose	Case–control study
Zhang, Z. 2023	US	10,052	2005–2016	MEC subsample weight (WTMEC2YR for 2005–2016)	Ethnicity, sex, poverty, marital, education, smoking, BMI, obesity, AST, ALT, and GGT	Cross-sectional study
Soltanieh, S. 2023	Iran	200	/	FFQ	Age, sex, blood glucose, smoking, physical activity, energy intake, BMI, WHtR, triglyceride, cholesterol, and HOMA	Cross-sectional study
Ramírez-Vélez, R. 2022	US	4,189	2017–2018	A review of the literature published up to 2010 linking diet and inflammatory markers	Sex, age, ethnicity, citizenship status, energy intake, alcohol consumption, smoking, physical activity, hypertension, HDL, obesity, and blood glucose	Cross-sectional study
Tyrovolas, S. 2019	Greece	2,992	2001–2012	FFQ	Age, sex, education, hypertension, blood glucose, cholesterol, obesity, and smoking	Cohort study
Valibeygi, A. 2023	Iran	9,792	Nov. 2014–Jun. 2019	FFQ	/	Cross-sectional study

No., number; FFQ, food frequency questionnaire; MEC, Mobile Exam Center; NHANES, National Health and Nutrition Examination Survey; BMI, body mass index; SES, socioeconomic status; HDL, high density lipoprotein cholesterol; LDL, low-density lipoprotein; TG, triglycerides; AST, aspartate aminotransferase; ALT, alanine aminotransferase; GGT, γ-glutamyl transferase; WHtR, waist to height ratio; HOMA, homeostasis model assessment.

Supplementary Table S1 shows the source of population, age at recruitment, median age and grouping subgroups of the different studies. Supplementary Table S2 (cohort study), Supplementary Table S3 (case–control study), and Supplementary Table S4 (cross-sectional study) show the evaluation quality of the 8 observational studies. Of these, 2 cohort studies scored 8 out of 10 for one case–control study scored 8 and the other scored 9 out of 10; and of the cross-sectional studies, 2 studies were 7 out of 10, one study scored 9 out of 10, and the other scored 10 out of 10. Collective, the quality of the included studies is high in general.

### Quantitative analysis

**Figure 2:**
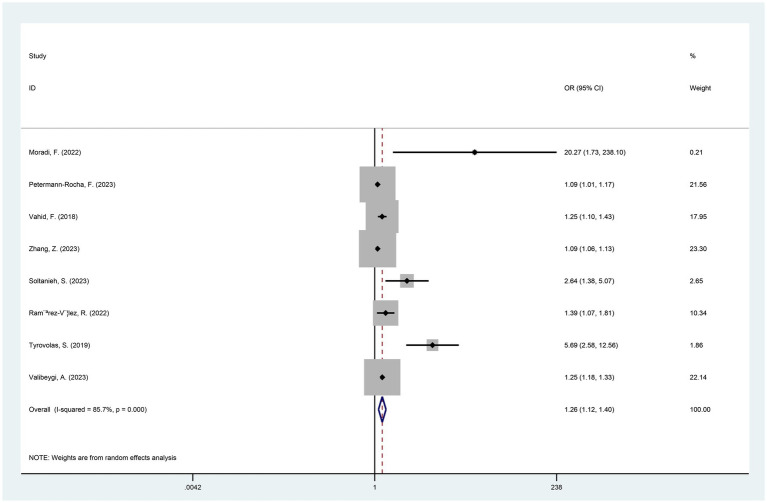
Forest plot of NAFLD risk induced by exposure to higher versus lower DII (*p* < 0.001).

**Figure 3:**
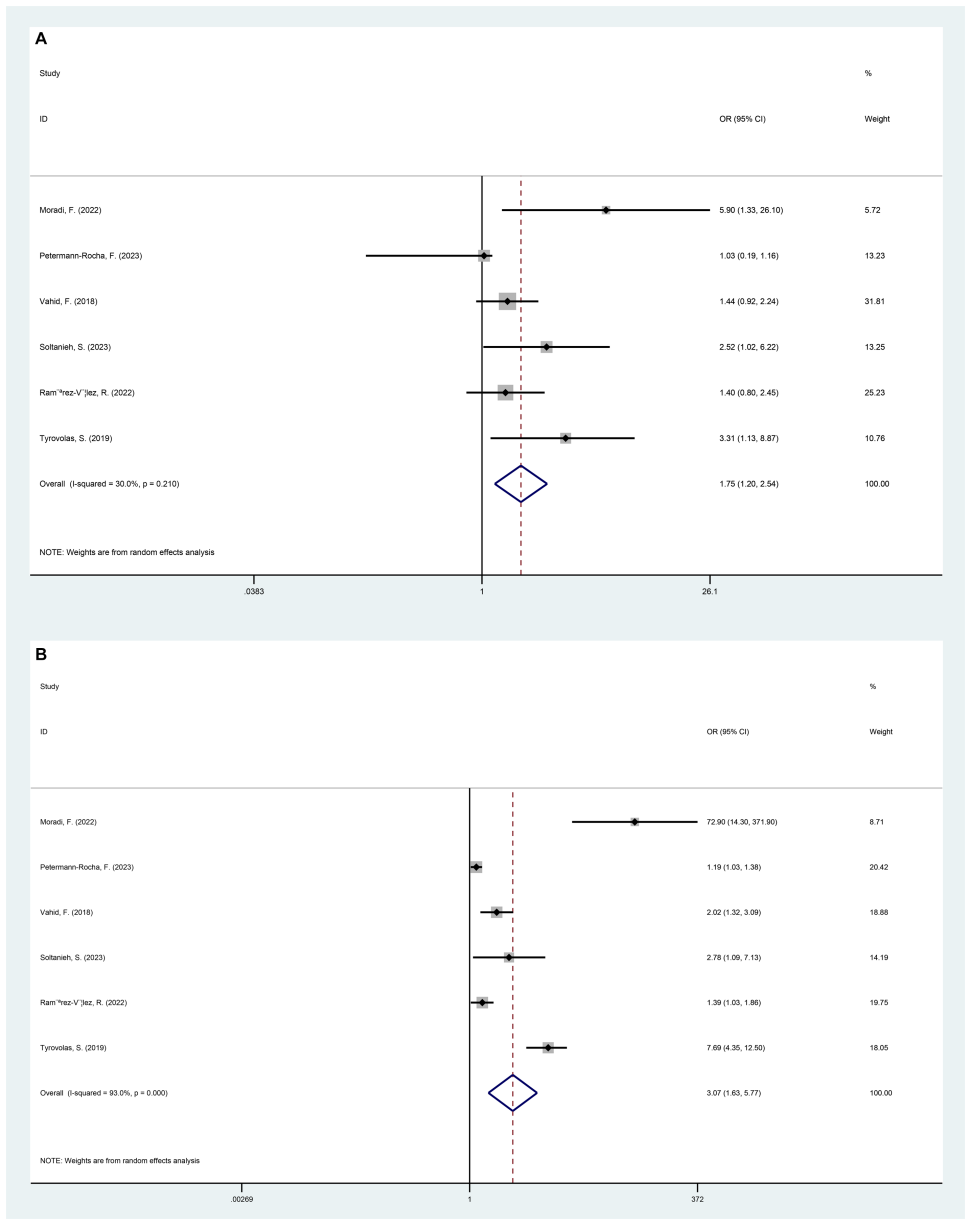
Forest plot of NAFLD risk induced by exposure to T1 versus T2/T3 by categorizing DII from low to high into different tertiles of T1, T2, and T3. **(A)** T1 versus T2 (*p* = 0.004); **(B)** T1 versus T3 (*p* = 0.001).

In general, the results of pooling the 8 datasets indicated that the participants exposed to higher DII had a higher risk of developing NAFLD (OR = 1.26, 95%CI 1.12 to 1.40, *p* < 0.001) (Figure 2). At the same time, the heterogeneity among the included studies was assessed by the chi-square test, and the results showed a high degree of heterogeneity (I^2^ = 85.7%, *p* < 0.001) (Figure 2). Potential heterogeneity was assessed by subgroup analyses. The DII was divided into 3 different quartiles in ascending order, namely T1, T2 and T3. Compared with T1, the risk of NAFLD in the T2 population was higher (OR = 1.75, 95%CI 1.20 to 2.54, *p* < 0.005). The risk of NAFLD was significantly higher in T3 participants compared to T1 (OR = 3.07, 95%CI 1.63 to 5.77, *p* = 0.001) (Figure 3).

In addition, when populations were classified by region (Table 2), there was no statistically significant difference in the risk of NAFLD among those affected by different DII in Europe, Iran and the US (Europe: OR = 2.37, 95%CI 0.47 to 11.94; Iran: OR = 2.25, 95%CI 0.92 to 5.49; US: OR = 1.19, 95%CI 0.94 to 1.49). By gender (Table 2), there was no obvious difference in the risk of NAFLD with different DII in the male population (OR = 2.64, 95%CI 0.66 to 10.56). However, for the female population, those exposed to higher DII had a higher risk of NAFLD (OR = 1.26, 95%CI 1.08 to 1.47). Secondly, among those with hypertension (Table 2), there was no statistical difference between participants with different DII (OR = 0.85, 95%CI 0.31 to 2.36). Meanwhile, in the smoking population (Table 2), those with high DII exposure had a higher risk of NAFLD (OR = 1.20, 95%CI 1.05 to 1.37). Finally, in the data we have obtained, when DII was the same, the male had a higher risk of NAFLD than females (OR = 1.11, 95%CI 1.00 to 1.24, *p* = 0.053).

**Table 2:** Subgroup analysis of the association between dietary inflammatory index and nonalcoholic fatty liver disease.

Subgroup	No. of studies	OR	95%CI	*p*	I^2^ (%)
Regional					
Europe	2	2.37	[0.47, 11.94]	0.295	94.0
Iran	4	2.25	[0.92, 5.49]	0.075	79.3
US	2	1.19	[0.95, 1.49]	0.140	69.1
Sex					
Male	2	2.64	[0.66, 10.56]	0.169	83.3
Female	2	1.26	[1.08, 1.47]	0.003	0.0
Other influencing factors					
Hypertension	2	0.85	[0.31, 2.36]	0.758	93.1
Smoking	2	1.20	[1.05, 1.37]	<0.001	34.3
Male versus Female	2	1.11	[1.00, 1.24]	0.053	0.0

No., number; OR, odds ratio; 95%CI, 95% confidence intervals.

### Sensitivity analysis and publication bias

To evaluate the effect of publication bias, the relationship between higher and lower DII in NAFLD was evaluated using Begg’s test (Supplementary Figure S1). Overall, there was no significant publication bias among studies (*p* > 0.1). Finally, sensitivity analyses were used to evaluate the stability of the results, where each study was excluded individually and was pooled for stability.

## Discussion

To our knowledge, there has been no previous meta-analysis of the relationship between DII and NAFLD been published so far. Our meta-analysis found a possible correlation between the risk of DII and NAFLD based on relevant cohort studies. The results suggested that people exposed to higher levels of DII may have a higher risk of NAFLD (OR = 1.26, 95%CI 1.12 to 1.40, *p* < 0.001). Correspondingly, the lower the exposure to DII, the lower the risk of NAFLD in the population.

Previous studies have shown that diet could affect NAFLD indirectly or directly by causing changes in inflammation in the body in various ways.

Pro-inflammatory diets, such as high-fat diets, can lead to the accumulation of ectopic lipids that cause the recruitment of macrophages (M1) and pro-inflammatory cytokines. It also activates inflammatory pathways in the brain by activating M1 macrophages and upregulating circulating pro-inflammatory cytokines (28). In conclusion, high-fat diets can cause oxidative stress in the body by activating a large number of signaling pathways. The level of inflammatory response in the body can have a direct impact on liver health. Pro-inflammatory diets may genetically interfere with hepatic β-oxidation, resulting in a massive upregulation of pro-inflammatory molecules (e.g., IL-1β, IL-17, and IL-18) and oxygen-responsive substances, which in turn increases the production of endogenous lipids and increases the risk of NAFLD, as well as exacerbating the damage related to chronic disease (1, 29, 30).

Anti-inflammatory diets, such as those rich in vegetables and fruits, are thought to be effective in preventing and ameliorating NAFLD (31). It contains flavonoids, α-tocopherol (vitamin E) and vitamin C which act as antioxidants to reduce oxidative stress in the body (32). The results of a randomized double-blind placebo-controlled trial showed that α-tocopherol (vitamin E) and vitamin C are excellent and potent antioxidants with anti-inflammatory properties (33). In addition, flavonoids have been reported extensively to be potent antioxidants in the prevention of NAFLD (34, 35). Their protective nature is attributed to their ability to accelerate the oxidation of fatty acids in the liver, enhance the antioxidant capacity of the cells and inhibit the production of nuclear factor-κ B, which in turn attenuates the release of inflammatory cytokines (36). As a result, it can reduce the risk of NAFLD through the mechanisms described above.

In addition, diet plays a crucial role in the regulation of inflammation levels in the body by influencing the maintenance of a balance of gut flora in the gut (37). Plant-based dietary patterns that have a lower potential to stimulate inflammation have been associated with the production of short-chain fatty acids (SCFA), a byproduct of gut flora, which have anti-inflammatory capacity. SCFA can affect energy metabolism and immunity by mediated via binding to G-protein coupled receptors expressed in the immune system and endocrine cells of the gut. In this way, SCFA can reduce the level of inflammation in the body (1, 38). Conversely pathogen-associated molecular proteins (PAMPs), products of intestinal microbes, also can increase inflammation levels in the liver. Recent studies in mice have shown that PAMPs, as a danger-associated molecular pattern (DAMPs), activate the hepatocyte inflammasome (a multiprotein cytoplasmic complex) in hepatocytes. Activation of inflammasomes in hepatocytes may be important for hepatocytes to cause initial stress, which subsequently leads to hepatocyte death and fibrosis (39–41).

Diets can also modulate inflammation levels through metabolic syndrome (MetS), including obesity and diabetes (42), which can lead to further development of NAFLD. A cross-sectional study of middle-aged and older adults found that higher DII was associated with a higher risk of MetS (43). Therefore, we guess that a high DII dietary pattern may indirectly increase the risk of NAFLD by increasing the risk of MetS.

In the case of obesity, for example, the state of obesity is often considered to be a causative factor of inflammation in the body (44), but there is growing evidence that inflammation can also contribute to the development of obesity (45). A longitudinal study of the MD showed that the higher the DII score in the exposed population, the progressively higher the incidence rate of overweight and obesity (46). Thus, pro-inflammatory diets trigger obesity by raising inflammation levels in the body. Obesity is an important risk factor for NAFLD. Hepatocytes in obese patients are exposed to high lipid and carbohydrate levels, leading to hepatic dysfunction and liver injury through dual processes of lipotoxicity and glucotoxicity (7, 30, 47).

Other MetS (including hypertension, hyperuric acid, and hyperlipidemia) have a bi-directional relationship with NAFLD, and these disorders can not only increase the risk of NAFLD but also increase some features and complications of these disorders (29, 48).

The genetic and environmental factors are known to influence the onset and development of NAFLD in individuals (49). A meta-analysis showed that the risk of NAFLD was lower in the black population and higher in the Hispanic population (50). In this regard, we analyzed subgroups of the population from different regions. However, our results were not statistically significant (Europe: OR = 2.37, 95%CI 0.47 to 11.94; Iran: OR = 2.25, 95%CI 0.92 to 5.49; US: OR = 1.19, 95%CI 0.94 to 1.49). This may be due to insufficient data on the subgroups in the included studies and the possibility that the same area contains multiple races.

Data from previous studies have shown that men have a significantly higher risk of developing NAFLD than women (51), and our results of subgroup analysis are consistent with these results (OR = 1.11, 95%CI 1.00 to 1.24). It suggests that gender is also an influential factor in NAFLD development. Estrogen may be a major contributor to the above phenomenon. A prospective study in Italy included 5,408 women with hysterectomies who were randomly assigned to tamoxifen (an estrogen inhibitor) or placebo. The results showed that tamoxifen was associated with an increased risk of NAFLD, especially in obese women (52). Additionally, we found that a pro-inflammatory diet increased the risk of NAFLD in the female population (OR = 1.26, 95%CI 1.08 to 1.47). This may be related to the fact that pro-inflammatory diets are associated with the modulation of inflammation levels in the body. A cross-sectional study of adult women in the United States found that a pro-inflammatory diet led to a decrease in sex hormone binding globulin (SHBG) (53). It has also been suggested that pro-inflammatory diets may be associated with decreased testosterone and estradiol levels in adolescent males (54). However, our findings were not statistically significant in the male population (OR = 2.64, 95%CI 0.66 to 10.56), which may be related to the paucity of relevant studies. More well-designed clinical trials are still needed to validate this aspect.

Our subgroup results suggest that pro-inflammatory diets increase the risk of NAFLD in chronic smokers (OR = 1.20, 95%CI 1.05 to 1.37). During smoking, nicotine accumulates in large quantities in gastric juices and saliva, producing pathogenic effects (55). On the one hand, cytotoxic chemicals produced by smoking may accelerate the growth and proliferation of fibroblasts, leading to the formation of scar tissue (56). On the other hand, smoking may also induce a massive recruitment of pro-inflammatory factors (IL-1, IL-6, and TNF-α) involved in hepatocyte injury (57). In addition, nicotine strongly stimulates hepatic injury and hepatic fibrosis by activating the nicotinic acetylcholine receptor (nAChR) in the liver (58). Smoking can increase the risk of NAFLD through multiple pathways along with pro-inflammatory diets.

Because of the high degree of heterogeneity among the included studies, for this, we performed subgroup analyses. Based on the results of the subgroups, we speculate that the different percentages of smoking, hypertension, and female characteristics in the included study populations may contribute to the potential heterogeneity. In addition, the different ethnicity and dietary habits of the sources of the included study population are also an important cause of heterogeneity. Finally, the differences in study design could also be an objective reason for the high degree of heterogeneity.

This study systematically explored the relationship between DII and NAFLD with some subgroup analysis. Meanwhile, our meta-analysis proves that the inflammatory potential of the diet might positively correlate with the risk of NAFLD. Additionally, the results of this meta-analysis can be used in clinical practice and public health interventions that aim to reduce the risk of NAFLD. Finally, our review may provide some reference for future clinical and basic trials on the relevant topics. There are also some limitations in this study. Due to the small sample size, more subgroup analysis could not be performed or the results were inaccurate. At the same time, the small number of studies included and the presence of a high degree of heterogeneity in this meta-analysis may affect the credibility of the results. Well-designed, larger clinical trials are required to prove this point.

## Conclusion

In conclusion, the results of our meta-analysis suggest an association between DII and the risk of NAFLD. The more pro-inflammatory the diet, the higher the risk of NAFLD; conversely, the more anti-inflammatory the diet, the lower the risk of NAFLD. Therefore, promoting foods low in DII could help reduce the risk of NAFLD in the population. In addition, more and further well-designed, large, multicenter clinical studies are needed to confirm the correlation between DII and NAFLD.

## Data availability statement

The original contributions presented in the study are included in the article/Supplementary material, further inquiries can be directed to the corresponding author.

## Author contributions

XZ: Conceptualization, Data curation, Formal analysis, Software, Supervision, Validation, Visualization, Writing – original draft, Writing – review & editing. JR: Data curation, Formal analysis, Resources, Software, Supervision, Validation, Writing – original draft, Writing – review & editing. YH: Data curation, Formal analysis, Resources, Software, Writing – original draft, Writing – review & editing. AX: Resources, Software, Supervision, Writing – original draft, Writing – review & editing. YF: Data curation, Supervision, Writing – original draft, Writing – review & editing. QZ: Conceptualization, Data curation, Writing – original draft, Writing – review & editing. LG: Formal analysis, Investigation, Writing – original draft, Writing – review & editing. XL: Formal analysis, Investigation, Methodology, Project administration, Resources, Writing – original draft, Writing – review & editing.

## Supplementary material

SUPPLEMENTARY FIGURE S1 Begg’s funnel plot with pseudo 95% confidence limits (*p*=0.108).

SUPPLEMENTARY FIGURE S2 Sensitivity analysis.

## Glossary

DII: dietary inflammation index
NAFLD: non-alcoholic fatty liver disease
TNF-α: tumor necrosis factor-α
IL-6: interleukin-6
IL-1β: interleukin-1β
MD: Mediterranean diet
PRISMA: the Preferred Reporting Items for Systematic Reviews and Meta-Analyses
RCT: randomized controlled trials
AHRQ: the Agency for Healthcare Research and Quality
OR: odds ratio
CI: confidence interval
FFQ: food frequency questionnaire
BMI: body mass index
NAFL: nonalcoholic fatty liver
NASH: nonalcoholic steatohepatitis
AASLD: the American Association for the Study of Liver Diseases
EASL: the European Association for the Study of the Liver
ALEH: the Latin American Association for the study of the liver
MASLD: metabolic dysfunction-associated steatotic liver disease
T1: Tertile 1
T2: Tertile 2
T3: Tertile 3
SCFA: short-chain fatty acids
PAMPs: pathogen-associated molecular proteins
DAMPs: danger-associated molecular pattern
MetS: metabolic syndrome
SHBG: sex hormone binding globulin
nAChR: nicotinic acetylcholine receptor
