# Supplementary figures and images for: Dietary inflammatory index and the risks of non-alcoholic fatty liver disease: a systematic review and meta-analysis
Source: Front Nutr. 2024 Jul 25;11:1388557. doi: 10.3389/fnut.2024.1388557 (PMC11309030; doi:10.3389/fnut.2024.1388557)

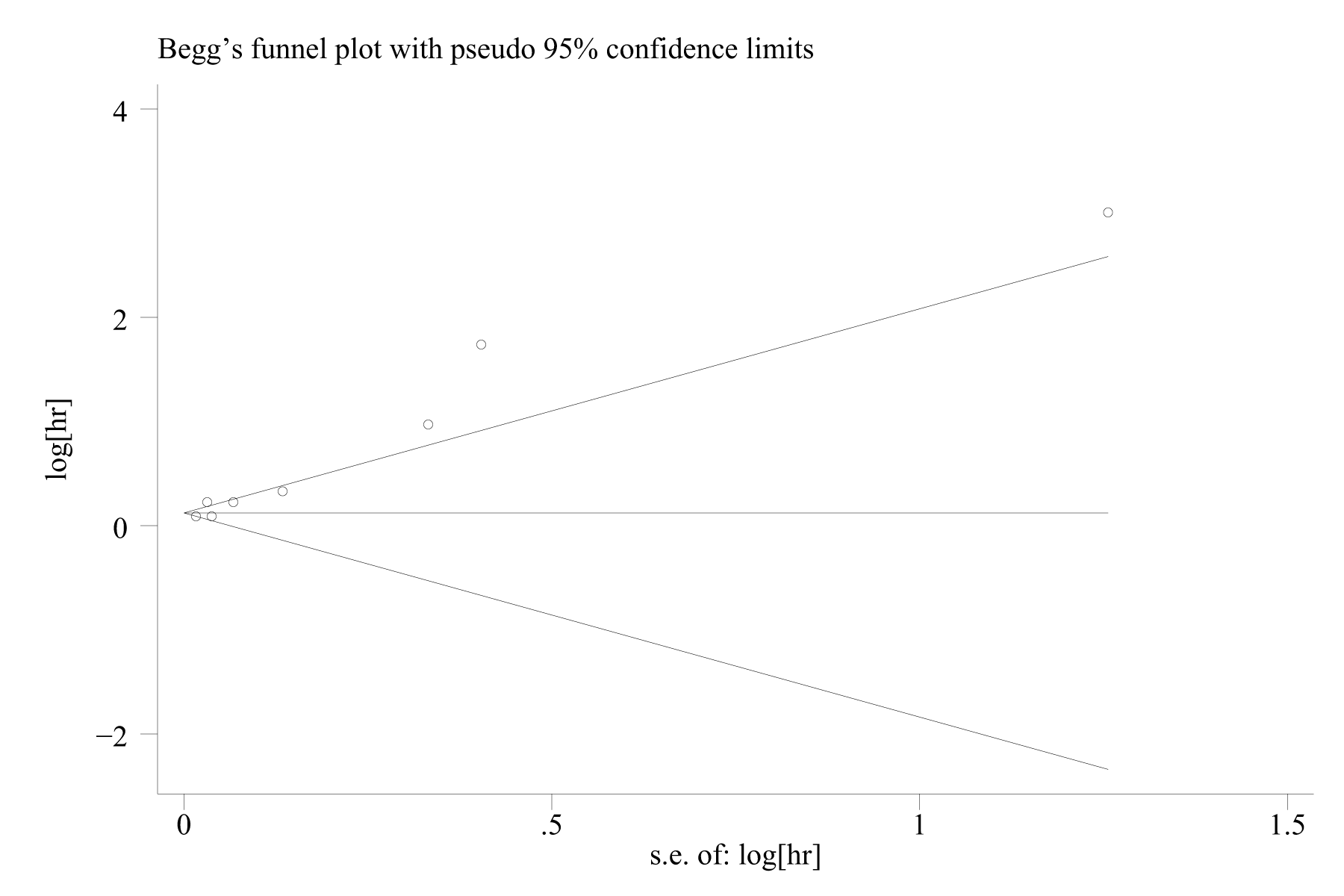

Supplement: SUPPLEMENTARY FIGURE S1 — Begg’s funnel plot with pseudo 95% confidence limits (p=0.108). [file Image_1.TIF]

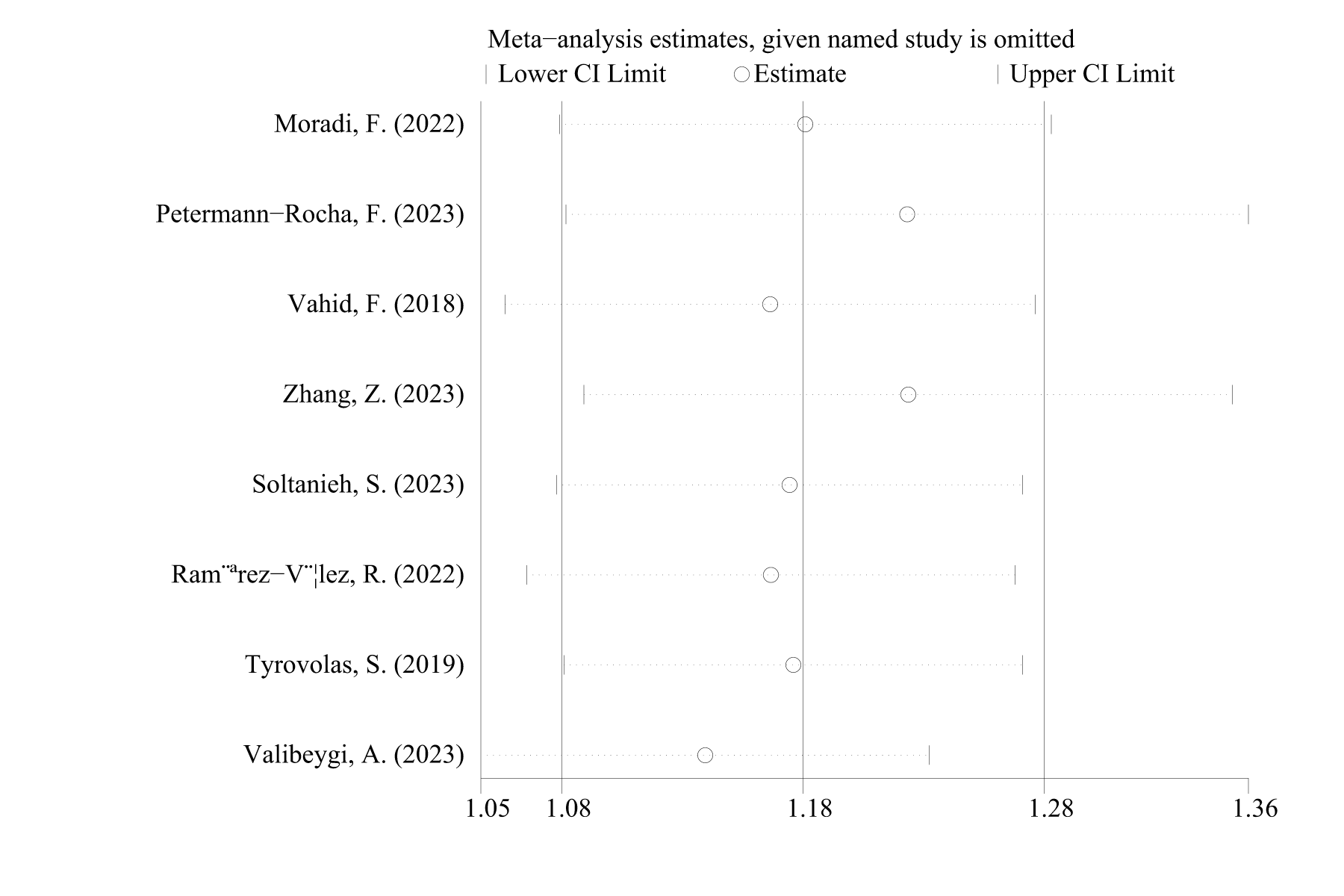

Supplement: SUPPLEMENTARY FIGURE S2 — Sensitivity analysis. [file Image_2.TIF]
