# Supplementary material for: Dietary inflammatory index and the risks of non-alcoholic fatty liver disease: a systematic review and meta-analysis
Source: Front Nutr. 2024 Jul 25;11:1388557. doi: 10.3389/fnut.2024.1388557 (PMC11309030; doi:10.3389/fnut.2024.1388557)
Supplement: Supplementary file 3 [file Table_1.DOCX]

**Supplementary Table 1**. Characteristics of included observational studies in the meta-analysis

| Author, year | Source of population | Age at recruitment | Median age at time of analysis (years) | Grouping of DII |
| --- | --- | --- | --- | --- |
| Moradi, F. 2022 | recruited from the Hospital | 18-50 | 36.8 | NA |
| Petermann-Rocha, F. 2023 | UK Biobank webpage | 37-73 | 56.1 | T1（-4.39~-1）；T2（-1~1）；T3（1~3.45） |
| Vahid, F. 2018 | specialized centers in Tehran | NA | 43.5 | T1（-4.44~-1.94）；T2（-1.94~-0.87）；T3（0.87~3.92） |
| Zhang, Z. 2023 | NHANES | NA | 46.1 | T1（-4.63~0.27）；T2（0.27~-1.80）；T3（1.80~3.03）；T4（3.03~5.47） |
| Soltanieh, S. 2023 | Institute of  Diabetes and Metabolism, Iran University of Medical Sciences | 18-70 | 52.2 | n=66,T1（<-3.11）；n=67,T2（-3.11~-2.49）；n=67,T3（>-2.49） |
| Ramírez-Vélez, R. 2022 | NHANES | 0-80 | 50.9 | n=1396,T1（-1.531）；n=1397,T2（0.226）；n=1396,T3（1.593） |
| Tyrovolas, S. 2019 | HNSSCS | NA | 45.0 | NA |
| Valibeygi, A. 2023 | Fasa PERSIAN cohort study | 35-70 | 48.7 | NA |

DII: Dietary Inflammatory Index; NA: not available; HNSSCS:Hellenic National Statistical Service Census Survey; NHANES: National Health and Nutrition Examination Survey; T1:Tertile 1; T2:Tertile 2; T3:Tertile 3; T4:Tertile 4.
