# Supplementary material for: Dietary inflammatory index and the risks of non-alcoholic fatty liver disease: a systematic review and meta-analysis
Source: Front Nutr. 2024 Jul 25;11:1388557. doi: 10.3389/fnut.2024.1388557 (PMC11309030; doi:10.3389/fnut.2024.1388557)
Supplement: Supplementary file 4 [file Table_2.DOCX]

**Supplementary Table 2.** Quality assessment of cohort studies included.

| Author, year | **Selection (Out of 4)** | | | | **Comparability**  **(Out of 2)** | **Outcomes (Out of 3)** | | | **Total**  **(Out of 9)** |
| --- | --- | --- | --- | --- | --- | --- | --- | --- | --- |
|  | Representativeness of exposed cohort | Selection of non exposed cohort | Ascertainment  of exposure | Outcome not present at the start of the study |  | Assessment of outcomes | Length of follow-up | Adequacy of follow up of cohorts |  |
| Petermann-Rocha, F. 2023 | 1 | 1 | 1 | 1 | 2 | 1 | 1 | 0 | 8 |
| Tyrovolas, S. 2019 | 1 | 1 | 1 | 1 | 1 | 1 | 1 | 1 | 8 |

The cohort studies were assessed by the Newcastle-Ottawa Quality Assessment Scale (NOS) checklist.
