# Supplementary material for: Dietary inflammatory index and the risks of non-alcoholic fatty liver disease: a systematic review and meta-analysis
Source: Front Nutr. 2024 Jul 25;11:1388557. doi: 10.3389/fnut.2024.1388557 (PMC11309030; doi:10.3389/fnut.2024.1388557)
Supplement: Supplementary file 5 [file Table_3.DOCX]

**Supplementary Table 3.** Quality assessment of case-control studies included.

| Author, year | **Selection (Out of 4)** | | | | **Comparability**  **(Out of 2)** | **Outcomes (Out of 3)** | | | **Total**  **(Out of 9)** |
| --- | --- | --- | --- | --- | --- | --- | --- | --- | --- |
|  | Adequate case definition | Representativeness of the cases | Selection of controls | Definition of controls |  | Ascertainment of exposure | Same method of ascertainment for cases and controls | Non-response rate |  |
| Moradi, F. 2022 | 1 | 1 | 1 | 1 | 2 | 1 | 1 | 1 | 9 |
| Vahid, F. 2018 | 1 | 1 | 1 | 1 | 1 | 1 | 1 | 1 | 8 |

The case-control studies were assessed by the Newcastle-Ottawa Quality Assessment Scale (NOS) checklist.
