# Supplementary material for: Dietary inflammatory index and the risks of non-alcoholic fatty liver disease: a systematic review and meta-analysis
Source: Front Nutr. 2024 Jul 25;11:1388557. doi: 10.3389/fnut.2024.1388557 (PMC11309030; doi:10.3389/fnut.2024.1388557)
Supplement: Supplementary file 6 [file Table_4.DOCX]

**Supplementary Table 4.** Quality assessment of cross-sectional studys included.

| **Item Yes/No/Unclear** | **Author, year** | | | |
| --- | --- | --- | --- | --- |
|  | Zhang, Z. 2023 | Soltanieh, S. 2023 | Ramírez-Vélez, R. 2022 | Valibeygi, A. 2023 |
| Define the source of information (survey, record review) | yes | yes | yes | yes |
| List inclusion and exclusion criteria for exposed and unexposed subjects (cases and controls) or refer to previous publications | yes | yes | yes | yes |
| Indicate time period used for identifying patients | yes | yes | yes | yes |
| Indicate whether or not subjects were consecutive if not population-based | unclear | no | unclear | yes |
| Indicate if evaluators of subjective components of study were masked to other aspects of the status of the participants | unclear | yes | yes | yes |
| Describe any assessments undertaken for quality assurance purposes (e.g., test/retest of primary outcome measurements) | yes | yes | yes | yes |
| Explain any patient exclusions from analysis | yes | yes | no | yes |
| Describe how confounding was assessed and/or controlled | yes | yes | yes | yes |
| If applicable, explain how missing data were handled in the analysis | no | yes | no | no |
| Summarize patient response rates and completeness of data collection | yes | yes | yes | yes |
| Clarify what follow-up, if any, was expected and the percentage of patients for which incomplete data or follow-up was obtained | unclear | yes | no | no |
| **Result** | 7 | 10 | 7 | 9 |

The cross-sectional studies were assessed by the Agency for Healthcare Research and Quality (AHRQ)checklist.
